# CT radiomics to differentiate neuroendocrine neoplasm from adenocarcinoma in patients with a peripheral solid pulmonary nodule: a multicenter study

**DOI:** 10.3389/fonc.2024.1420213

**Published:** 2024-06-17

**Authors:** Xiaoyu Liu, Hongjian Li, Shengping Wang, Shan Yang, Guobin Zhang, Yonghua Xu, Hanfeng Yang, Fei Shan

**Affiliations:** ^1^ Department of Radiology, Shanghai Public Health Clinical Center, Fudan University, Shanghai, China; ^2^ Department of Radiology, Affiliated Hospital of North Sichuan Medical College, North Sichuan Medical College, Nanchong, China; ^3^ Department of Radiology, Fudan University Shanghai Cancer Center, Fudan University, Shanghai, China; ^4^ Department of Radiology, Zhongshan Hospital, Fudan University, Shanghai, China; ^5^ Department of Radiology, Shanghai Sixth People’s Hospital Affiliated to Shanghai Jiao Tong University School of Medicine, Shanghai Jiao Tong University, Shanghai, China; ^6^ Department of Imaging and Interventional Radiology, Zhongshan-Xuhui Hospital of Fudan University, Fudan University, Shanghai, China

**Keywords:** radiomics, lung neuroendocrine neoplasm, lung adenocarcinoma, peripheral solid pulmonary nodule, tomography, X-ray computed

## Abstract

**Purpose:**

To construct and validate a computed tomography (CT) radiomics model for differentiating lung neuroendocrine neoplasm (LNEN) from lung adenocarcinoma (LADC) manifesting as a peripheral solid nodule (PSN) to aid in early clinical decision-making.

**Methods:**

A total of 445 patients with pathologically confirmed LNEN and LADC from June 2016 to July 2023 were retrospectively included from five medical centers. Those patients were split into the training set (n = 316; 158 LNEN) and external test set (n = 129; 43 LNEN), the former including the cross-validation (CV) training set and CV test set using ten-fold CV. The support vector machine (SVM) classifier was used to develop the semantic, radiomics and merged models. The diagnostic performances were evaluated by the area under the receiver operating characteristic curve (AUC) and compared by Delong test. Preoperative neuron-specific enolase (NSE) levels were collected as a clinical predictor.

**Results:**

In the training set, the AUCs of the radiomics model (0.878 [95% CI: 0.836, 0.915]) and merged model (0.884 [95% CI: 0.844, 0.919]) significantly outperformed the semantic model (0.718 [95% CI: 0.663, 0.769], *p* both<.001). In the external test set, the AUCs of the radiomics model (0.787 [95% CI: 0.696, 0.871]), merged model (0.807 [95%CI: 0.720, 0.889]) and semantic model (0.729 [95% CI: 0.631, 0.811]) did not exhibit statistical differences. The radiomics model outperformed NSE in sensitivity in the training set (85.3% vs 20.0%; *p* <.001) and external test set (88.9% vs 40.7%; *p* = .002).

**Conclusion:**

The CT radiomics model could non-invasively, effectively and sensitively predict LNEN and LADC presenting as a PSN to assist in treatment strategy selection.

## 1 Introduction

Lung neuroendocrine neoplasm (LNEN) encompasses a spectrum of tumors that originate from pulmonary neuroendocrine cells, including small cell lung cancer (SCLC), large cell neuroendocrine carcinoma and carcinoid tumor. LNEN accounts for approximately 20% of pulmonary primary malignant tumors and its incidence is constantly increasing (1, 2). However, lung adenocarcinoma (LADC) as the predominant histological type, mainly arises from the alveolar epithelial cells of small bronchial mucosa, representing approximately 40% of pulmonary primary malignant tumors (3, 4). LADC is often treated with surgery and early-stage cases even could be cured by lobectomy. Moreover, segmentectomy is recommended for LADC with diameter ≤ 2cm (5, 6). However, LNEN, particularly in poorly differentiated cases with rapid growth, often demonstrates heightened metastatic potential upon detection, leading to more advanced stage of the disease and less benefit from surgery or localized treatment (1, 7–9). For early-stage patients with LNEN detected on chest computed tomography (CT) scans, surgical resection is recommended after ruling out distant metastasis though positron emission tomography/computed tomography and brain magnetic resonance imaging and confirming negative mediastinal lymph nodes on pathology (10–14). Furthermore, lobectomy is preferred over sublobectomy (14). Consequently, the different biological behaviors of LNEN and LADC significantly impact treatment strategies and prognosis, and early diagnosis is crucial to guide treatment and improve prognosis.

CT, as the preferred method for chest diseases, plays a crucial role in non-invasive diagnosis in lung cancer. In the clinic, LNEN typically presents as a central mass with rapid growth, while LADC often manifests as a peripheral nodule with different ground-glass component. In contrast to the typical manifestations, LNEN appearing as a peripheral solid nodule (PSN) is exceedingly rare and shares similar radiological findings with LADC. Moreover, both LNEN and LADC, manifesting as a PSN, are primarily observed in the early stages and typically lack associated clinical symptoms or signs (15). Therefore, the preoperative differential diagnosis of LNEN and LADC appearing as a PSN is quite challenging. Although radiologists could distinguish LNEN from LADC by analyzing their CT radiological findings to some extent, but the evaluation of radiological findings is subjective and prone to interobserver variation (16). Additionally, preoperative serum neuron-specific enolase (NSE) is also a prevalent tumor marker for non-invasive clinical prediction of LNEN. However, its predictive power is limited due to its relatively low sensitivity, ranging from 30% to 72.5% (17–20).

Radiomics, a non-invasive, quantitative and objective prediction method, can extract feature information from digital images to assist in clinical decision-making (21–23). Previous studies have demonstrated that radiomics could effectively differentiate between LNEN and other cancers (24–26). However, research concerning the differential diagnosis of peripheral LNEN and LADC is scarce, with existing studies being conducted at a single center and lacking independent external validation (19, 27). Therefore, the objective of this study was to develop a radiomics model using preoperative chest thin-section non-contrast CT to discriminate LNEN from LADC presenting as a PSN. Subsequently, independent external validation was performed to further explore its robust and generalization.

## 2 Materials and methods

The institutional review boards of five participating centers (Zhongshan Hospital [center 1], Shanghai Sixth People’s Hospital Affiliated to Shanghai Jiao Tong University School of Medicine [center 2], Zhongshan-Xuhui Hospital of Fudan University [center 3], Fudan University Shanghai Cancer Center [center 4], Affiliated Hospital of North Sichuan Medical College [center 5]) approved this retrospective multicenter study. Written informed consent was waived for the retrospective nature of this study.

### 2.1 Study patients

**Figure 1:**
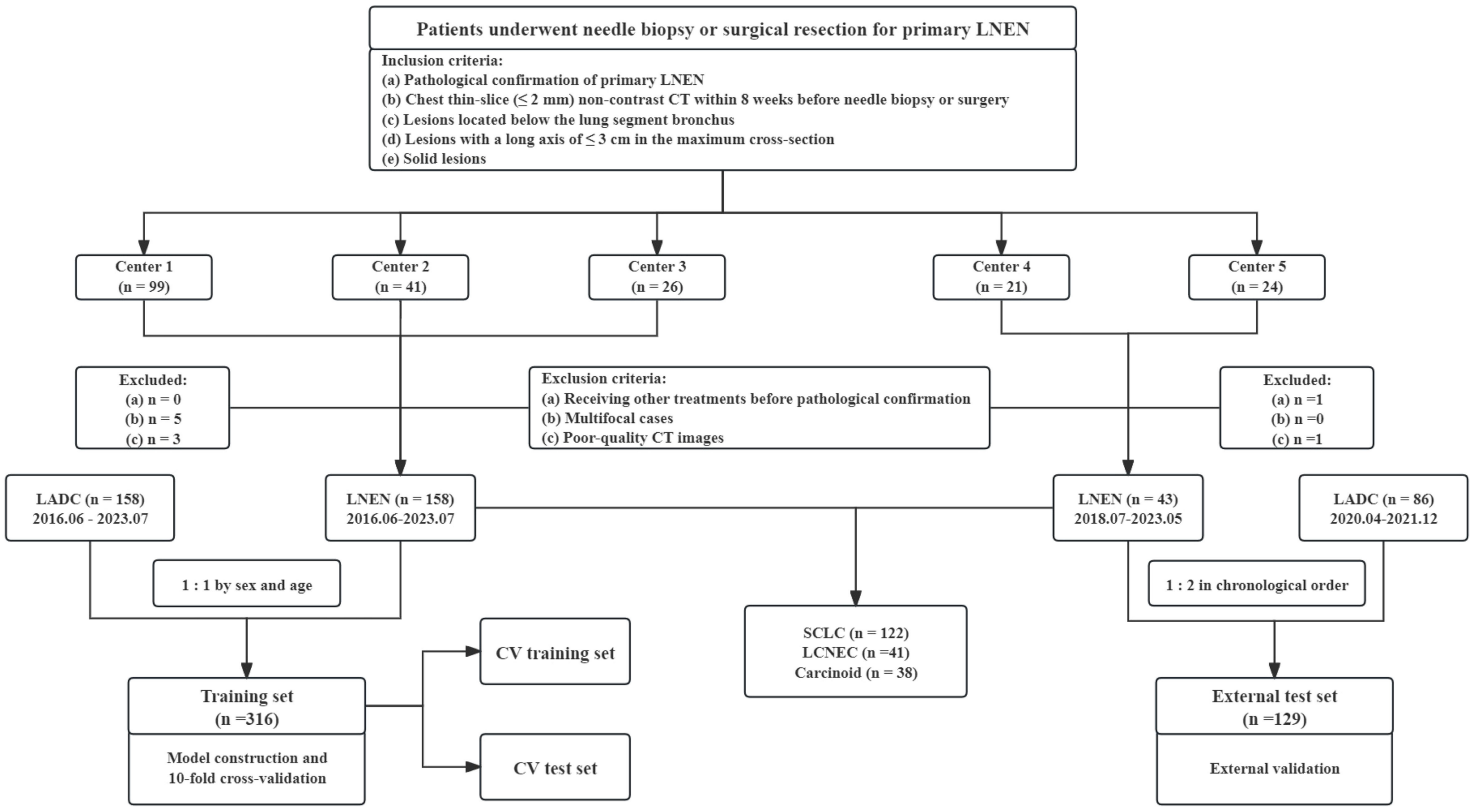
Flow diagram of the patient selection from five medical centers. LNEN, lung neuroendocrine neoplasm; LADC, lung adenocarcinoma; CV, cross-validation; SCLC, small cell lung cancer; LCNEC, large cell neuroendocrine carcinoma. Center 1 indicates Zhongshan Hospital, Center 2 indicates Shanghai Sixth People’s Hospital Affiliated to Shanghai Jiao Tong University School of Medicine, Center 3 indicates Zhongshan-Xuhui Hospital of Fudan University, Center 4 indicates Fudan University Shanghai Cancer Center, Center 5 indicates Affiliated Hospital of North Sichuan Medical College.

Patients from five medical centers who underwent needle biopsy or surgical resection for primary LNEN (between June 2016 and July 2023) were considered for this retrospective study. The inclusion criteria were as follows: (a) pathological confirmation of primary LNEN, (b) chest thin-slice (≤ 2mm) non-contrast CT within eight weeks before needle biopsy or surgery, (c) lesions located below the lung segment bronchus, (d) lesions with a long axis of ≤ 3cm in the maximum cross-section, (e) solid lesions. The exclusion criteria were as follows: (a) receiving other treatments before pathological confirmation, (b) multifocal cases, (c) poor-quality CT images. The detailed process of recruitment is presented in **Figure 1**.

Patients from center 1–3 comprised the training set for model training and internal validation, while those in center 4 and 5 served as the external test set for external validation. The inclusion and exclusion criteria for LADC were the same as those for LNEN, except for the pathological diagnosis. Due to the predominance of male cases in LNEN, LADC patients in center 1–3 included in the training set were matched 1:1 by sex and age to minimize differences between groups and better train models. To evaluate the generalization of models in proximity to the real world, LADC cases twice as many as LNEN were chronologically collected in the external test set from center 4 and 5. Collection process was stopped once the number of LADC cases reached twice that of LNEN. Data collection spanned from July 2018 to May 2023 at center 4 and 5.

### 2.2 CT study protocols

Patients underwent chest thin-slice (slice thickness ranging from 1.00 to 2.00 mm) non-contrast CT within 8 weeks before needle biopsy or surgery. Detailed imaging protocols are explained in **Supplementary Table S1**.

### 2.3 Clinical characteristics and radiological signs assessment

**Figure 2:**
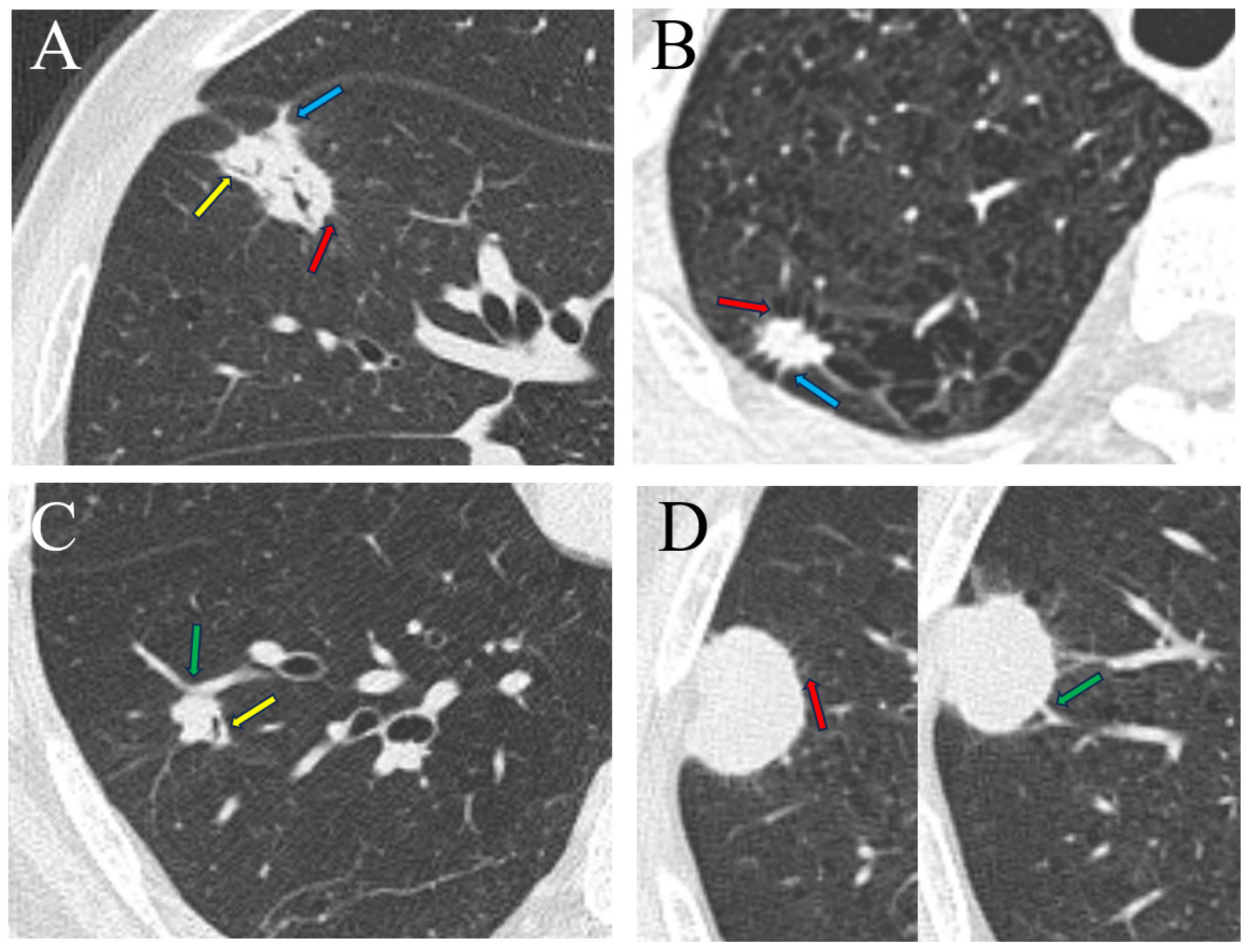
Radiological signs of four types of lung tumor. **(A)** A 74-year-old man with lung adenocarcinoma in the medial segment of the middle lobe of the right lung, exhibiting signs of lobulation, spiculation, air bronchogram, and pleural indentation. **(B)** A 58-year-old man with lung large cell neuroendocrine carcinoma in the apical segment of the upper lobe of the right lung, displaying lobulation, spiculation and pleural indentation. **(C)** A 58-year-old man with lung carcinoid tumor in the anterior basal segment of the lower lobe of the right lung, demonstrating lobulation, air bronchogram, and vascular convergence sign. **(D)** An 81-year-old man with small cell lung cancer in the posterior segment of the upper lobe of the right lung, presenting signs of lobulation, spiculation, and vascular convergence sign. Spiculation (red arrow), air bronchogram (yellow arrow), pleural indentation (blue arrow), vascular convergence sign (green arrow).

Clinical data, including sex, age, and preoperative NSE levels (if available), were collected from the electronic medical record system. The NSE levels were standardized into dichotomous variables by a cutoff value of 16.30 ng/ml to be a clinical predictor for LNEN (NSE level ≥ 16.3 ng/ml). The volumes of interest (VOIs) were firstly automatically segmented by a deep learning network provided by the commercial software uAI Research Portal (United Imaging Intelligence Co., Ltd, China) (28). Subsequently, these VOIs were successively checked by a junior radiologist (XYL, with 2 years of experience in chest imaging) and a senior radiologist (FS, with 22 years of experience in chest imaging) and corrected if necessary. The radiological signs were initially evaluated by XYL and then reviewed by FS. The seven evaluated radiological signs (**Figure 2**) were as follows: (a) outer 1/3 lung zone, (b) upper lobe of right lung, (c) lobulation, (d) spiculation, (e) pleural indentation, (f) air bronchogram, (g) vascular convergence sign. The outer 1/3 lung zone refers to dividing each lung into three equal parts using concentric circles starting from the hilus and selecting the outermost third of the lung, which is another method for differentiating central from peripheral types.

### 2.4 Radiomics feature extraction

To minimize noise interference and normalize the background information prior to imaging, we transformed the grayscale images using a window level of -600 HU and a window width of 1200 HU. The image voxel dimensions were resampled to 1×1×1 mm (x-, y-, and z-axes), with an voxel array shift of 1000 and an image discretization bin width of 25. The open-source Python package PyRadiomics (version 3.1.0; http://pyradiomics.readthedocs.io/) was employed to extract radiomics features from chest thin-slice non-contrast CT. We explored eight types of images for feature extraction: Original; Wavelet; LoG with sigma values of 1, 2, 3, 4, 5; Square; SquareRoot; Logarithm; Exponential; Gradient. Each type of images was extracted with seven types of features: shape-based; first-order; second-order: grey level cooccurrence matrix (GLCM), grey level dependence matrix (GLDM), grey level size zone matrix (GLSZM), grey level run length matrix (GLRLM), neighborhood gray-tone difference matrix (NGTDM). In total, 1781 features were extracted for per patient.

### 2.5 Model development

**Figure 3:**
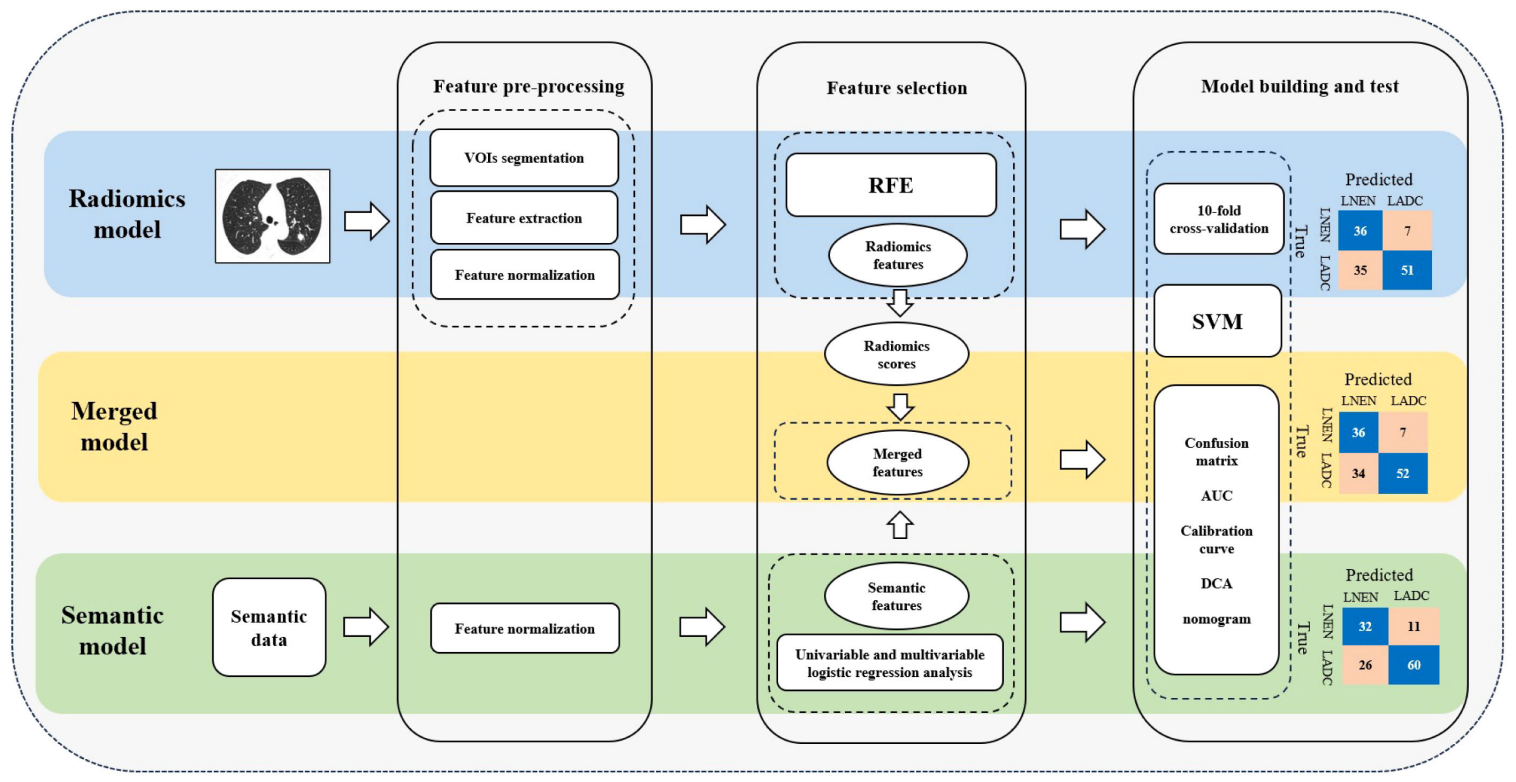
Workflow for feature pre-processing, feature selection and model building. LNEN, lung neuroendocrine neoplasm; LADC, lung adenocarcinoma; VOIs, volumes of interest; RFE, recursive feature elimination; SVM, support vector machine; AUC, area under the receiver operating characteristic curve; DCA, decision curve analysis.

The model construction process, illustrated in **Figure 3**, employed the open-source Python package scikit-learn (version 0.24.2; https://scikit-learn.org/stable/) for data processing and model construction. Firstly, each extracted feature underwent Z-score normalization to ensure comparability. Secondly, the recursive feature elimination (RFE) method was applied for feature selection. Subsequently, the training set was split into cross-validation (CV) training set and CV test set by the ten-fold CV method to train and internally validate the radiomics model based on the SVM classifier. The trained optimal model parameters obtained from ten-fold CV were then fitted to the training set to check for overfitting. Additionally, the radiomics model was externally validated in the external test set to evaluate its generalization.

In the training set, univariable and multivariable logistic regression analysis were employed to identify independent risk-factors of LNEN and LADC among standardized radiological signs. These risk-factors were then used to develop a semantic model. The merged model incorporated the radiological signs applied in the semantic model and radiomics scores from the radiomics model to investigate whether the combination of radiological signs and radiomics information can improve predictive performance. Additionally, SVM classifier and ten-fold CV were utilized in both the construction and internal validation of the semantic model and merged model. External validation of both these two models were performed in the external test set.

### 2.6 Statistical analyses

Continuous variables were presented as medians and interquartile ranges (IQR), analyzed using the Mann-Whitney U test for group comparisons. Categorical variables were presented as frequencies and percentages, and their group comparisons were conducted by Pearson’s chi-squared test or McNemar test. Univariable and multivariable logistic regression analysis were conducted to identify risk-factors with odds ratio (OR) and 95% confidence interval (CI). A nomogram was constructed for the merged model. The model performance was evaluated using the area under the receiver operating characteristic curve (AUC) and compared using the Delong method. For the cases with NSE levels, the McNemar test was also used to compare the diagnostic performance metrics (e.g., accuracy, sensitivity, specificity) of the radiomics model and NSE in distinguishing LNEN from LADC. Calibration curve was plotted to compare the predicted values with the observed values. Decision curve analysis was used to assess clinical utility. Statistical analysis was performed with Python (version 3.9.12; https://www.python.org/), R software (version 4.2.2; https://www.r-project.org/) and SPSS software (version 25.0). A two-sided P value less than 0.05 was considered statistically significant.

## 3 Results

### 3.1 Patients characteristics

Among the 201 patients with primary LNEN, 122 cases were SCLC, 41 cases were large cell neuroendocrine carcinoma and 38 cases were carcinoid tumor. Additionally, 244 patients with primary LADC were included in this study. A total of 445 patients (median age, 64 years [IQR, 57–69 years]; 345 men) were included, with 316 (158 LNEN) in the training set and 129 (43 LNEN) in the external test set. Furthermore, among the 445 patients included in this study, 254 patients had NSE examinations (median age, 64 years [IQR, 58–69 years]; 189 men): 161 (75 LNEN) in the training set and 93 (27 LNEN) in the external test set. All baseline characteristics are detailed in **Table 1**; **Supplementary Table S2**.

**Table 1:** Baseline patient characteristics in the training set and external test set.

Characteristic	Training Set (n=316)		P value	External Test Set (n=129)		P value
	LNEN (n=158)	LADC (n=158)		LNEN (n=43)	LADC (n=86)	
Age (y) ^†^	65.0 (60.0, 69.0)	65.0 (60.0, 69.0)	NA	58.0 (53.0, 66.0)	64.0 (56.0, 71.0)	.025^*^
Sex (male)	131 (82.9)	131 (82.9)	NA	34 (79.1)	49 (57.0)	.014^*^
Outer 1/3 lung zone (present)	77 (48.7)	104 (65.8)	.005^*^	14 (32.6)	49 (57.0)	.009^*^
RU (present)	49 (31.0)	47 (29.7)	.897	18 (41.9)	22 (25.6)	.060
Lobulation (present)	139 (88.0)	157 (99.4)	<.001^*^	38 (88.4)	85 (98.8)	.008^*^
Spiculation (present)	34 (21.5)	77 (48.7)	<.001^*^	10 (23.3)	39 (45.3)	.015^*^
Pleural indentation (present)	29 (18.4)	68 (43.0)	<.001^*^	5 (11.6)	39 (45.3)	<.001^*^
Air bronchogram (present)	2 (1.3)	20 (12.7)	<.001^*^	1 (2.3)	11 (12.8)	.108
Vascular convergence sign (present)	53 (33.5)	62 (39.2)	.380	10 (23.3)	32 (37.2)	.111

Unless otherwise indicated, data are numbers of patients, and data in parentheses are percentages. LNEN, lung neuroendocrine neoplasm; LADC, lung adenocarcinoma; RU, upper lobe of right lung; NA, not applicable.

^†^Data are medians, with interquartile ranges in parentheses.

^*^P-values are statistically significant.

Compared with the LADC group, the LNEN group exhibited significantly lower occurrences in the outer 1/3 lung zone, lobulation, spiculation, and pleural indentation in both the training set and external test set (*p* <.05 for all) (**Table 1**). However, the statistical difference of air bronchogram was only observed in the training set (*p* <.001), but not in the external test set (*p* = .108). There was no statistical difference in the seven radiological signs between the training set and external test set (**Supplementary Table S3**).

### 3.2 Model construction

**Figure 4:**
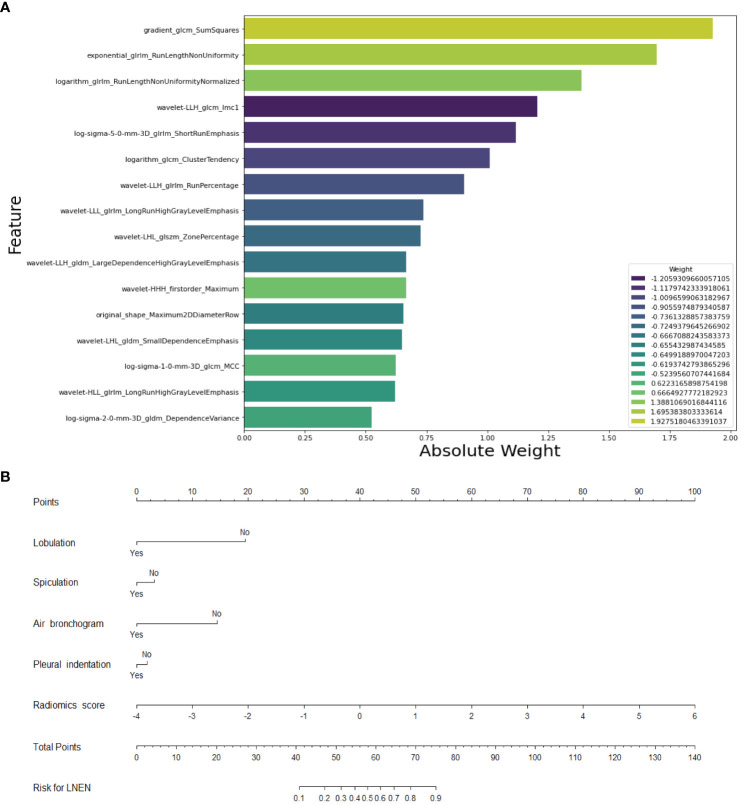
Selected Features for the construction of the radiomics model and merged model. **(A)** Feature weight map of the radiomics model. **(B)** Nomogram of the merged model for differentiating neuroendocrine neoplasm from adenocarcinoma in patients with a peripheral solid pulmonary nodule. LNEN, lung neuroendocrine neoplasm.

Three models were developed in this study to distinguish LNEN from LADC in patients with a PSN: a radiomics model, a semantic model and a merged model. Sixteen radiomics features (14 second-order features, 1 first-order feature, 1 shape-based feature) filtered by RFE were applied to build a radiomics model employing the SVM classifier (**Figure 4A**). The LNEN group exhibited higher radiomics scores than the LADC group in both the training (median, 0.982 [IQR: 0.392, 1.824] vs -1.000 [IQR: -1.682, -0.328]; *p* <.001) and external test set (median, 1.246 [IQR: 0.438, 2.102] vs -0.227 [IQR: -1.169, 0.467]; *p* <.001) (**Supplementary Figure S1**).

Univariable and multivariable logistic regression analysis conducted in the training set revealed that lobulation (OR = 0.099 [95% CI: 0.013, 0.765]; *p* = .027), spiculation (OR = 0.440 [95% CI: 0.255, 0.759]; *p* = .003), pleural indentation (OR = 0.516 [95% CI: 0.286, 0.930]; *p* = .028), and air bronchogram (OR = 0.109 [95% CI: 0.025, 0.488]; *p* = .004) were independent risk-factors (**Table 2**). The semantic model utilized lobulation, spiculation, pleural indentation and air bronchogram to build an SVM model. These four radiological signs were then combined with the radiomics scores to create an SVM-based merged model (**Figure 4B**).

**Table 2:** Logistic regression analysis of variables for their association with LNEN and LADC in the training set.

Characteristic	Univariable Analysis	P value	Multivariable Analysis	P value
	OR		OR	
Outer 1/3 lung zone (present *vs* absent)	0.494 (0.314, 0.777)	.002^*^	0.693 (0.412, 1.164)	.166
RU (present vs absent)	1.062 (0.657, 1.715)	.807	NA	NA
Lobulation (present vs absent)	0.047 (0.006, 0.353)	.003^*^	0.099 (0.013, 0.765)	.027^*^
Spiculation (present vs absent)	0.288 (0.176, 0.471)	<.001^*^	0.440 (0.255, 0.759)	.003^*^
Pleural indentation (present vs absent)	0.298 (0.178, 0.496)	<.001^*^	0.516 (0.286, 0.930)	.028^*^
Air bronchogram (present vs absent)	0.088 (0.020, 0.385)	.001^*^	0.109 (0.025, 0.488)	.004^*^
Vascular convergence sign (present vs absent)	0.782 (0.494, 1.237)	.293	NA	NA

Data in parentheses are 95% CIs. LNEN, lung neuroendocrine neoplasm; LADC, lung adenocarcinoma; OR, odds ratio; RU, upper lobe of right lung; NA, not applicable.

^*^P-values are statistically significant.

### 3.3 Performance of models and NSE for differentiating LNEN from LADC

In the ten-fold CV analysis in the training set, the radiomics model and merged model had higher AUCs than the semantic model (**Table 3**). The semantic model, radiomic model and merged model recorded AUCs of 0.707 (95% CI: 0.648, 0.762), 0.879 (95% CI: 0.836, 0.919) and 0.887 (95% CI: 0.845, 0.925) in the CV training set, respectively. In the CV test set, AUCs were 0.708 (95% CI: 0.531, 0.863) for the semantic model, 0.852 (95% CI: 0.699, 0.972) for the radiomics model and 0.878 (95% CI: 0.738, 0.983) for the merged model. The optimal model parameters derived from the ten-fold CV were implemented on the training set without overfitting for all three models.

**Table 3:** Mean AUCs and accuracies of models in CV training set and CV test set.

	Semantic model		Radiomics model		Merged model	
	AUC ^†^	Accuracy (%)	AUC ^†^	Accuracy (%)	AUC ^†^	Accuracy (%)
CV Training Set	0.707 (0.648, 0.762)	68.3	0.879 (0.836, 0.919)	82.9	0.887 (0.845, 0.925)	84.1
CV Test Set	0.708 (0.531, 0.863)	67.1	0.852 (0.699, 0.972)	81.0	0.878 (0.738, 0.983)	83.9

Unless otherwise indicated, data are the means derived from the 10-fold cross-validation. AUC, area under the receiver operating characteristic curve; CV, cross validation.

^†^Data in parentheses are 95% CIs.

**Figure 5:**
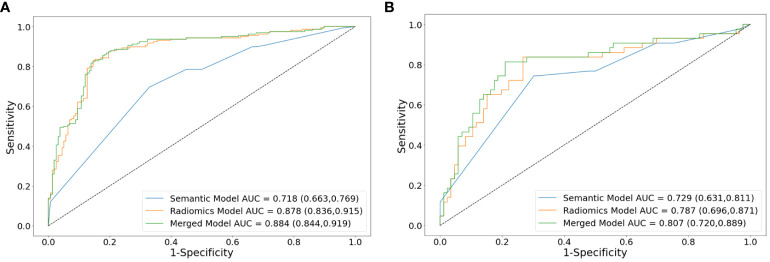
Receiver operating characteristic curve analysis of models for differentiating lung neuroendocrine neoplasm from adenocarcinoma in the training set **(A)** and external test set **(B)**. AUCs are reported with 95%CIs in parentheses. AUC, area under the receiver operating characteristic curve.

**Figure 6:**
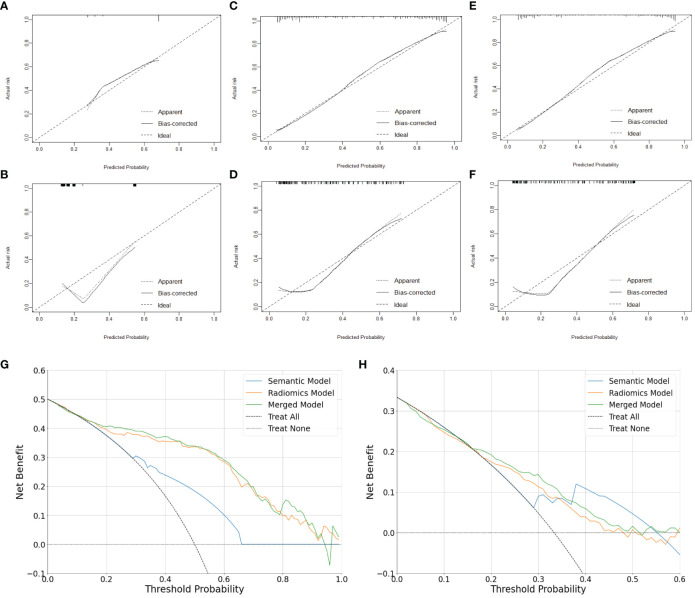
Calibration and clinical utility of the models for differentiating lung neuroendocrine neoplasm from adenocarcinoma. Calibration curves of the semantic model **(A, B)**, radiomics model **(C, D)** and merged model **(E, F)** in the training set and external test set, respectively. Decision Curves of the models in the training set **(G)** and external test set **(H)**, respectively.

In the training set, the AUCs of both the radiomics model (0.878 [95% CI: 0.836, 0.915]; *p* <.001) and merged model (0.884 [95% CI: 0.844, 0.919]; *p* <.001) significantly outperformed the semantic model (0.718 [95% CI: 0.663, 0.769]). However, the AUCs of both the radiomics model (0.787 [95% CI: 0.696, 0.871], *p* = .351) and merged model (0.807 [95% CI: 0.720, 0.889], *p* = .183) did not exhibit statistical differences compared to the semantic model (0.729 [95% CI: 0.631, 0.811]) in the external test set. The performance of all the models is shown in **Table 4**. The receiver operating characteristic curves, calibration curves and clinical decision curves are provided in **Figures 5**, **6**. The calibration curves showed the radiomics model with the best performance between the predicted probability and the actual probability. Decision curves showed that three models could achieve net benefit within a reasonable range of threshold probabilities.

**Table 4:** Diagnostic performance of models for differentiating LNEN from LADC.

	AUC ^†^	Accuracy (%)	Sensitivity (%)	Specificity(%)	PPV (%)	NPV (%)	P value ^‡^
Semantic model							
Training Set	0.718 (0.663, 0.769)	68.4 (216/316)	69.6 (110/158)	67.1 (106/158)	67.9 (110/162)	68.8 (106/154)	Ref
External Test Set	0.729 (0.631, 0.811)	71.3 (92/129)	74.4 (32/43)	69.8 (60/86)	55.2 (32/58)	84.5 (60/71)	Ref
Radiomics model							
Training Set	0.878 (0.836, 0.915)	83.9 (265/316)	83.5 (132/158)	84.2 (133/158)	84.1 (132/157)	83.6 (133/159)	<.001^*^
External Test Set	0.787 (0.696, 0.871)	67.4 (87/129)	83.7 (36/43)	59.3 (51/86)	50.7 (36/71)	87.9 (51/58)	.351
Merged model							
Training Set	0.884 (0.844, 0.919)	84.2 (266/316)	82.9 (131/158)	85.4 (135/158)	85.1 (131/154)	83.3 (135/162)	<.001^*^
External Test Set	0.807 (0.720, 0.889)	68.2 (88/129)	83.7 (36/43)	60.5 (52/86)	51.4 (36/70)	88.1 (52/59)	.183

Unless otherwise indicated, data are percentages, with proportions of patients(numerator/denominator) in parentheses. Ref, reference; LNEN, lung neuroendocrine neoplasm; LADC, lung adenocarcinoma; AUC, area under the receiver operating characteristic curve; PPV, positive predictive value; NPV, negative predictive value.

^†^Data in parentheses are 95% CIs.

^‡^P value was calculated with the Delong test and indicates the significance level of the comparison of AUCs with the semantic model as the reference in the corresponding data set.

^*^P-values are statistically significant.

Notably, in 254 patients (102 LNEN) with NSE examination, the radiomics model demonstrated superior sensitivity than NSE in both the training set (64 of 75 [85.3%] vs 15 of 75 [20.0%]; *p* <.001) and external test set (24 of 27 [88.9%] vs 11 of 27 [40.7%]; *p* = .002) for distinguishing between LNEN and LADC. The performance of the radiomics model and NSE is shown in **Table 5**.

**Table 5:** Diagnostic performance of the radiomics model and NSE.

Data Set	Radiomics model	NSE	P value
Training Set (n=161)			
Accuracy (%)	84.5 (136/161)	59.0 (95/161)	<.001^*^
Sensitivity (%)	85.3 (64/75)	20.0 (15/75)	<.001^*^
Specificity (%)	83.7 (72/86)	93.0 (80/86)	.118
External Test Set (n=93)			
Accuracy (%)	67.7 (63/93)	62.4 (58/93)	.575
Sensitivity (%)	88.9 (24/27)	40.7 (11/27)	.002^*^
Specificity (%)	59.1 (39/66)	71.2 (47/66)	.243

Unless otherwise indicated, data are percentages, with proportions of patients (numerator/denominator) in parentheses. NSE, neuron-specific enolase.

^*^P-values are statistically significant.

## 4 Discussion

The existing limited studies primarily focus on cases presenting with masses and the differential diagnosis of peripheral SCLC and LADC in single-center studies (19, 27). We developed and internally validated a radiomics model using preoperative chest thin-section non-contrast CT to discriminate LNEN from LADC manifested as a PSN and performed external validation to assess the performance of the model. The AUCs of the radiomics model were 0.878 in the training set and 0.787 in the external test set, respectively. Furthermore, in 254 patients with NSE examination, the radiomics model exceled NSE in sensitivity in both the training set (85.3% vs 20.0%, *p* <.001) and external test set (88.9% vs 40.7%, *p* = .002). The satisfactory predictive performance of the CT radiomics model implied its potential for non-invasively, quantitatively, objectively and sensitively discriminate between LNEN and LADC manifesting as a PSN, thereby aiding in treatment guidance.

Preoperative histological biopsy is a commonly used invasive method for identifying histological type of lung cancer when diagnosis is challenging. However, this method is invasive and highly dependent on the operators’ experience for successful diagnosis. Compared with the localized sampling of biopsy, CT screening non-invasively offers comprehensive information about the lesion. In our study, LNEN presenting as a PSN was less with lobulation, spiculation, pleural indentation and air bronchogram, which was consistent with previous studies on differential diagnosis of peripheral SCLC and LADC (19, 20, 29). This consistency possibly is attributed to the fact that LNEN all originates from pulmonary neuroendocrine cells and LNEN included in our study were predominantly SCLC. The semantic model developed by radiological findings in this study achieved AUCs of 0.718 and 0.729 in the training set and external test set, respectively, which indicated CT radiological findings could help differentiate LNEN from LADC appearing as a PSN to some extent. The differences of radiological findings between LNEN and LADC may be associated with the propensity of LADC to involve local regions and induce changes in surrounding pulmonary structures.

Radiomics is considered as a digital biopsy approach for predicting tumor biological characteristics (30–32). A previous study using a CT-based radiomics model successfully differentiated peripheral SCLC from LADC with AUCs yielding 0.858 and 0.836 in the training set and validation set, respectively (19). Our radiomics model based on preoperative chest thin-slice non-contrast CT displayed satisfactory performance in distinguishing between LNEN and LADC presenting as a PSN, with AUCs of 0.879 and 0.852 for the CV training set and CV test set, respectively. Furthermore, this radiomics model still achieved an acceptable AUC of 0.787 in the external test set. The 14 filtered second-order texture features (e.g., gradient-glcm-SumSquares, exponential-glrlm-RunLengthNonUniformity) of our radiomics model may potentially reflect the difference in the uniformity of lesion density (33), which might be related to the fact that SCLC exhibits greater homogeneity in comparison with LADC (20, 34). In addition, the radiomics method offered a quantitative and objective assessment approach, especially when combined with automatic three-dimensional segmentation rather than manual segmentation and two-dimensional segmentation (35–37). Therefore, the radiomics model could potentially mitigate misdiagnosis from inexperienced radiologists and enhance diagnostic reliability in comparison with the subjectivity and variability of the semantic model based on radiological signs evaluated by radiologists (38, 39). Additionally, the performance of the merged model had improved on the benchmark of the radiomics model, suggesting that radiological signs may enhance diagnostic performance to some extent (40), but further validation with a larger sample size remains necessary. Besides, the inclusion of manually evaluated radiological signs in the merged model also made it less convenient and objective than the radiomics model.

NSE, a commonly used clinical predictor for LNEN, demonstrated a sensitivity of 72.5% in a cohort of 80 peripheral SCLC cases, half of which were in advanced stages (19). However, this sensitivity decreased to 52.4% in a smaller cohort of 21 SCLC cases presenting as a peripheral nodule (20). Moreover, the sensitivity was only 39.2% in resectable lung carcinoid tumor (41). This suggests that NSE expression may increase with more advanced stages and higher-grade LNEN. Regrettably, only 254 patients (102 LNEN) in our study had NSE levels available, possibly due to the rarity of LNEN presenting as a PSN which leads clinicians to overlook it and not perform NSE examination. In this study, the sensitivity of NSE was notably low, only 20.0% for the training set and 40.7% for the external set, potentially due to the predominance of early-stage cases and the inclusion of lung carcinoid tumor cases. Compared with NSE, the radiomics model exhibited statistically significant superior sensitivity of 85.3% and 88.9% for the training set and external test set, respectively, across a cohort of 254 patients undergoing NSE testing. These findings suggest that the radiomics model offered a substantial improvement in suggesting LNEN over NSE, positioning it as a promising non-invasive predictive tool. Consequently, this radiomics model could facilitate subsequent positron emission tomography/computed tomography, brain magnetic resonance imaging and/or needle biopsy examination for clinical diagnosis and staging, guiding the selection of optimal treatment strategies.

Our study also had several limitations. Firstly, the retrospective nature of this study may induce selection bias, despite efforts have been made to match LNEN with LADC based on sex and age in the training set to minimize differences between groups, which may also affect models’ performance to some extent. Furthermore, prospective studies are necessary to validate the generalizability of our model. Secondly, the sample size in our study was relatively limited. Although we have collected 202 cases of peripherally LNEN data from five centers, a larger sample size is required for further validation and data-driven deep learning. Thirdly, enlargement of mediastinal or hilar lymph node was not included, as our study mainly focused on the characteristics of the nodule itself. Finally, the radiomics features in this study were solely extracted from unenhanced chest CT images. While chest non-contrast CT scans are straightforward and low-cost, further studies using chest enhanced CT images are needed to identify subtler invisible variations in uniformity of density, thereby improving diagnostic accuracy.

In conclusion, the CT radiomics model demonstrated effective performance in distinguishing between LNEN and LADC in patients with a PSN. Therefore, the radiomics model may serve as a non-invasive, quantitative, objective and sensitive approach for differentiating peripheral LNEN from LADC.

## Data availability statement

The raw data supporting the conclusions of this article will be made available by the authors, without undue reservation.

## Ethics statement

The studies involving humans were approved by Medical Ethics Committee of Shanghai Public Health Clinical Center. The studies were conducted in accordance with the local legislation and institutional requirements. The ethics committee/institutional review board waived the requirement of written informed consent for participation from the participants or the participants’ legal guardians/next of kin because this is a retrospective study.

## Author contributions

XL: Formal analysis, Investigation, Methodology, Project administration, Writing – original draft. HL: Formal analysis, Investigation, Methodology, Writing – original draft. SW: Investigation, Writing – review & editing. SY: Investigation, Writing – review & editing. GZ: Investigation, Writing – review & editing. YX: Investigation, Writing – review & editing. HY: Investigation, Writing – review & editing. FS: Conceptualization, Funding acquisition, Methodology, Writing – review & editing.

## References

[1] WHO Classification of Tumours Editorial Board. Thoracic tumours. Lyon (France: International Agency for Research on Cancer (2021).

[2] Dasari A Shen C Halperin D Zhao B Zhou S Xu Y. Trends in the incidence, prevalence, and survival outcomes in patients with neuroendocrine tumors in the United States. JAMA Oncol. (2017) 3:1335–42. doi: 10.1001/jamaoncol.2017.0589 PMC5824320 28448665

[3] Zhang Y Vaccarella S Morgan E Li M Etxeberria J Chokunonga E. Global variations in lung cancer incidence by histological subtype in 2020: a population-based study. Lancet Oncol. (2023) 24:1206–18. doi: 10.1016/s1470-2045(23)00444-8 37837979

[4] Debieuvre D Molinier O Falchero L Locher C Templement-Grangerat D Meyer N. Lung cancer trends and tumor characteristic changes over 20 years (2000–2020): Results of three French consecutive nationwide prospective cohorts’ studies. Lancet Reg Health Eur. (2022) 22:100492. doi: 10.1016/j.lanepe.2022.100492 36108315 PMC9445429

[5] Saji H Okada M Tsuboi M Nakajima R Suzuki K Aokage K. Segmentectomy versus lobectomy in small-sized peripheral non-small-cell lung cancer (JCOG0802/WJOG4607L): a multicentre, open-label, phase 3, randomised, controlled, non-inferiority trial. Lancet. (2022) 399:1607–17. doi: 10.1016/s0140-6736(21)02333-3 35461558

[6] Hattori A Suzuki K Takamochi K Wakabayashi M Sekino Y Tsutani Y. Segmentectomy versus lobectomy in small-sized peripheral non-small-cell lung cancer with radiologically pure-solid appearance in Japan (JCOG0802/WJOG4607L): a *post-hoc* supplemental analysis of a multicentre, open-label, phase 3 trial. Lancet Respir Med. (2024) 12:105–16. doi: 10.1016/S2213-2600(23)00382-X 38184010

[7] Kawasaki K Rekhtman N Quintanal-Villalonga Á Rudin CM. Neuroendocrine neoplasms of the lung and gastrointestinal system: convergent biology and a path to better therapies. Nat Rev Clin Oncol. (2023) 20:16–32. doi: 10.1038/s41571-022-00696-0 36307533

[8] Park HK Kwon GY. Comparison of metastatic patterns among neuroendocrine tumors, neuroendocrine carcinomas, and nonneuroendocrine carcinomas of various primary organs. J Korean Med Sci. (2023) 38:e85. doi: 10.3346/jkms.2023.38.e85 36942393 PMC10027546

[9] Filosso PL Oliaro A Ruffini E Bora G Lyberis P Asioli S. Outcome and prognostic factors in bronchial carcinoids: a single-center experience. J Thorac Oncol. (2013) 8:1282–8. doi: 10.1097/JTO.0b013e31829f097a 24457239

[10] Kalemkerian GP. Staging and imaging of small cell lung cancer. Cancer Imaging. (2012) 11:253–8. doi: 10.1102/1470-7330.2011.0036 PMC3266593 22245990

[11] Jett JR Schild SE Kesler KA Kalemkerian GP. Treatment of small cell lung cancer: Diagnosis and management of lung cancer, 3rd ed: American College of Chest Physicians evidence-based clinical practice guidelines. Chest. (2013) 143:e400S–e19S. doi: 10.1378/chest.12-2363 23649448

[12] Yu JB Decker RH Detterbeck FC Wilson LD. Surveillance epidemiology and end results evaluation of the role of surgery for stage I small cell lung cancer. J Thorac Oncol. (2010) 5:215–9. doi: 10.1097/JTO.0b013e3181cd3208 20101146

[13] Yang CJ Chan DY Shah SA Yerokun BA Wang XF D’Amico TA. Long-term survival after surgery compared with concurrent chemoradiation for node-negative small cell lung cancer. Ann Surg. (2018) 268:1105–12. doi: 10.1097/sla.0000000000002287 28475559

[14] National Comprehensive Cancer Network. NCCN clinical practice guidelines in oncology (NCCN guidelines): small cell lung cancer (2024). Available online at: https://www.nccn.org/patiens. 10.6004/jnccn.2204.0023 38754467

[15] Patel VK Naik SK Naidich DP Travis WD Weingarten JA Lazzaro R. A practical algorithmic approach to the diagnosis and management of solitary pulmonary nodules: part 1: radiologic characteristics and imaging modalities. Chest. (2013) 143:825–39. doi: 10.1378/chest.12-0960 23460160

[16] Liu C Zhao R Pang M. Semantic characteristic grading of pulmonary nodules based on deep neural networks. BMC Med Imaging. (2023) 23:156. doi: 10.1186/s12880-023-01112-4 37833636 PMC10571455

[17] Tsoli M Koumarianou A Angelousi A Kaltsas G. Established and novel circulating neuroendocrine tumor biomarkers for diagnostic, predictive and prognostic use. Best Pract Res Clin Endocrinol Metab. (2023) 37:101785. doi: 10.1016/j.beem.2023.101785 37336711

[18] Korse CM Taal BG Vincent A van Velthuysen ML Baas P Buning-Kager JC. Choice of tumour markers in patients with neuroendocrine tumours is dependent on the histological grade. A marker study of Chromogranin A, Neuron specific enolase, Progastrin-releasing peptide and cytokeratin fragments. Eur J Cancer. (2012) 48:662–71. doi: 10.1016/j.ejca.2011.08.012 21945100

[19] Wang J Zhong F Xiao F Dong X Long Y Gan T. CT radiomics model combined with clinical and radiographic features for discriminating peripheral small cell lung cancer from peripheral lung adenocarcinoma. Front Oncol. (2023) 13:1157891. doi: 10.3389/fonc.2023.1157891 37020864 PMC10069670

[20] Zhang X Lv F Fu B Li W Lin R Chu Z. Clinical and computed tomography characteristics for early diagnosis of peripheral small-cell lung cancer. Cancer Manag Res. (2022) 14:589–601. doi: 10.2147/cmar.S351561 35210856 PMC8857949

[21] Gillies RJ Kinahan PE Hricak H. Radiomics: images are more than pictures, they are data. Radiology. (2016) 278:563–77. doi: 10.1148/radiol.2015151169 PMC4734157 26579733

[22] Chen M Copley SJ Viola P Lu H Aboagye EO. Radiomics and artificial intelligence for precision medicine in lung cancer treatment. Semin Cancer Biol. (2023) 93:97–113. doi: 10.1016/j.semcancer.2023.05.004 37211292

[23] Cellina M Cè M Irmici G Ascenti V Khenkina N Toto-Brocchi M. Artificial intelligence in lung cancer imaging: unfolding the future. Diagnostics (Basel). (2022) 12:2644. doi: 10.3390/diagnostics12112644 36359485 PMC9689810

[24] Adelsmayr G Janisch M Müller H Holzinger A Talakic E Janek E. Three dimensional computed tomography texture analysis of pulmonary lesions: Does radiomics allow differentiation between carcinoma, neuroendocrine tumor and organizing pneumonia? Eur J Radiol. (2023) 165:110931. doi: 10.1016/j.ejrad.2023.110931 37399666

[25] Martini I Polici M Zerunian M Panzuto F Rinzivillo M Landolfi F. CT texture analysis of liver metastases in PNETs versus NPNETs: Correlation with histopathological findings. Eur J Radiol. (2020) 124:108812. doi: 10.1016/j.ejrad.2020.108812 31951893

[26] Liu S Liu S Zhang C Yu H Liu X Hu Y. Exploratory study of a CT radiomics model for the classification of small cell lung cancer and non-small-cell lung cancer. Front Oncol. (2020) 10:1268. doi: 10.3389/fonc.2020.01268 33014770 PMC7498676

[27] Chen BT Chen Z Ye N Mambetsariev I Fricke J Daniel E. Differentiating peripherally-located small cell lung cancer from non-small cell lung cancer using a CT radiomic approach. Front Oncol. (2020) 10:593. doi: 10.3389/fonc.2020.00593 32391274 PMC7188953

[28] Wu J Xia Y Wang X Wei Y Liu A Innanje A. uRP: An integrated research platform for one-stop analysis of medical images. Front Radiol. (2023) 3:1153784. doi: 10.3389/fradi.2023.1153784 37492386 PMC10365282

[29] Jiang B Takashima S Miyake C Hakucho T Takahashi Y Morimoto D. Thin-section CT findings in peripheral lung cancer of 3 cm or smaller: are there any characteristic features for predicting tumor histology or do they depend only on tumor size? Acta Radiol. (2014) 55:302–8. doi: 10.1177/0284185113495834 23926233

[30] Limkin EJ Sun R Dercle L Zacharaki EI Robert C Reuzé S. Promises and challenges for the implementation of computational medical imaging (radiomics) in oncology. Ann Oncol. (2017) 28:1191–206. doi: 10.1093/annonc/mdx034 28168275

[31] Dercle L Lu L Schwartz LH Qian M Tejpar S Eggleton P. Radiomics response signature for identification of metastatic colorectal cancer sensitive to therapies targeting EGFR pathway. J Natl Cancer Inst. (2020) 112:902–12. doi: 10.1093/jnci/djaa017 PMC7492770 32016387

[32] Tomaszewski MR Gillies RJ. The biological meaning of radiomic features. Radiology. (2021) 298:505–16. doi: 10.1148/radiol.2021202553 PMC7924519 33399513

[33] Zhou W Zhang L Wang K Chen S Wang G Liu Z. Malignancy characterization of hepatocellular carcinomas based on texture analysis of contrast-enhanced MR images. J Magn Reson Imaging. (2017) 45:1476–84. doi: 10.1002/jmri.25454 27626270

[34] Xu X Sui X Zhong W Xu Y Wang Z Jiang J. Clinical utility of quantitative dual-energy CT iodine maps and CT morphological features in distinguishing small-cell from non-small-cell lung cancer. Clin Radiol. (2019) 74:268–77. doi: 10.1016/j.crad.2018.10.012 30691731

[35] Gitto S Corino VDA Annovazzi A Milazzo MaChado E Bologna M Marzorati L. 3D vs. 2D MRI radiomics in skeletal Ewing sarcoma: Feature reproducibility and preliminary machine learning analysis on neoadjuvant chemotherapy response prediction. Front Oncol. (2022) 12:1016123. doi: 10.3389/fonc.2022.1016123 36531029 PMC9755864

[36] Rexha I Laage-Gaupp F Chapiro J Miszczuk MA van Breugel JMM Lin M. Role of 3D quantitative tumor analysis for predicting overall survival after conventional chemoembolization of intrahepatic cholangiocarcinoma. Sci Rep. (2021) 11:9337. doi: 10.1038/s41598-021-88426-x 33927226 PMC8085245

[37] Defeudis A Mazzetti S Panic J Micilotta M Vassallo L Giannetto G. MRI-based radiomics to predict response in locally advanced rectal cancer: comparison of manual and automatic segmentation on external validation in a multicentre study. Eur Radiol Exp. (2022) 6:19. doi: 10.1186/s41747-022-00272-2 35501512 PMC9061921

[38] Liu Y Liu W Chen H Xie S Wang C Liang T. Artificial intelligence versus radiologist in the accuracy of fracture detection based on computed tomography images: a multi-dimensional, multi-region analysis. Quant Imaging Med Surg. (2023) 13:6424–33. doi: 10.21037/qims-23-428 PMC10585498 37869340

[39] Obuchowicz R Oszust M Piorkowski A. Interobserver variability in quality assessment of magnetic resonance images. BMC Med Imaging. (2020) 20:109. doi: 10.1186/s12880-020-00505-z 32962651 PMC7509933

[40] Zhao J Sun Z Yu Y Yuan Z Lin Y Tan Y. Radiomic and clinical data integration using machine learning predict the efficacy of anti-PD-1 antibodies-based combinational treatment in advanced breast cancer: a multicentered study. J Immunother Cancer. (2023) 11:e006514. doi: 10.1136/jitc-2022-006514 37217246 PMC10230987

[41] Georgakopoulou VE Zygouris E Damaskos C Pierrakou A Papalexis P Garmpis N. Prognostic value of the immunohistochemistry markers CD56, TTF-1, synaptophysin, CEA, EMA and NSE in surgically resected lung carcinoid tumors. Mol Clin Oncol. (2022) 16:31. doi: 10.3892/mco.2021.2464 34984102 PMC8719249

