# Supplementary material for: CT radiomics to differentiate neuroendocrine neoplasm from adenocarcinoma in patients with a peripheral solid pulmonary nodule: a multicenter study
Source: Front Oncol. 2024 Jun 17;14:1420213. doi: 10.3389/fonc.2024.1420213 (PMC11215045; doi:10.3389/fonc.2024.1420213)
Supplement: Supplementary file 1 [file DataSheet_1.docx]

**
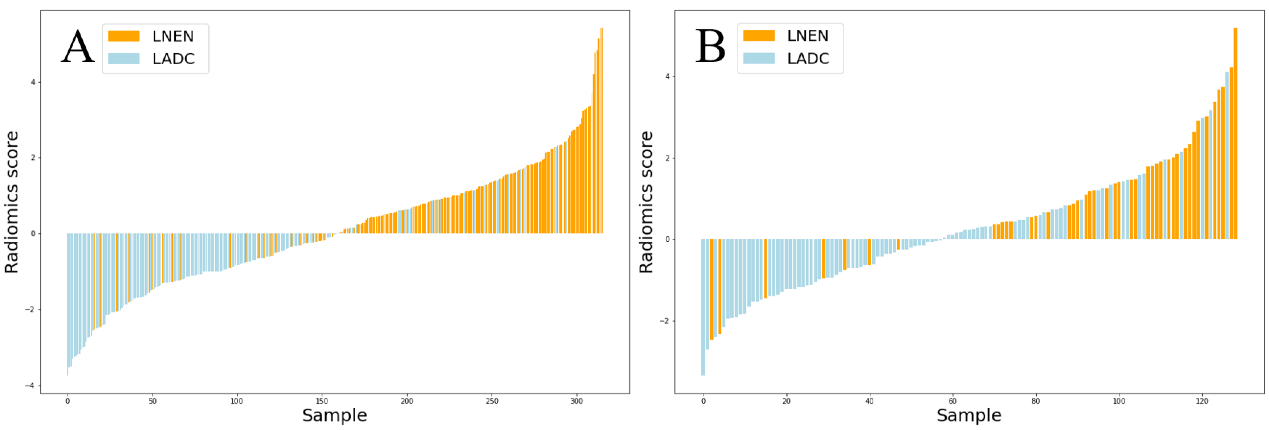
**

**Figure S1:** Waterfall plots show the radiomics scores of LNEN and LADC in the training set **(A)** and external test set **(B)**. LNEN = lung neuroendocrine neoplasm, LADC = lung adenocarcinoma.

**Table S1**  **Technical parameters of CT examination**

|  | Center 1 | Center 2 | Center 3 | Center 4 | Center 5 |
| --- | --- | --- | --- | --- | --- |
| Machine type | SIEMENS SOMATOM Emotion 16, go.All, Definition AS, Definition AS+; TOSHIBA Aquilion, Aquilion ONE;  UIH uCT 760, 780, 960+;  Philips Ingeuity CT; GE MEDICAL SYSTEMS LightSpeed VCT | GE MEDICAL SYSTEMS Revolution CT; SIEMENS SOMATOM Definition AS+, Force; Philips iCT 256, Brilliance 64 | SIEMENS Sensation 64; Philips Ingenuity CT, Brilliance 64; GE MEDICAL SYSTEMS Optima CT680; UIH uCT 780 | SIMENS SOMATOM Perspective, Definition Edge; TOSHIBA Aquilion ONE; GE MEDICAL SYSTEMS Revolution CT | UIH uCT 510, 710; Philips MX 16-slice; SIEMENS SOMATOM Definition Edge, Force; GE MEDICAL SYSTEMS LightSpeed VCT |
| Tube voltage (KV) | 120~130 | 120 | 120 | 120~130 | 120 |
| Tube current (MA) | automatic | automatic | automatic | automatic | automatic |
| Section thickness (mm) | 1.00, 1.25, 1.50 | 1.00, 1.25 | 1.00, 1.25, 1.50 | 1.00, 1.25 | 1.00, 1.25, 1.50, 2.00 |
| Section interval (mm) | 1.00, 1.25, 1.50 | 1.00, 1.25 | 1.00, 1.25, 1.50 | 1.00, 1.25 | 1.00, 1.25, 1.50, 2.00 |
| Matrix | 512🞨512 | 512🞨512 | 512🞨512 | 512🞨512 | 512🞨512 |
| Convolution algorithm | B70s, B70f, FC52, FC56, B_SHARP_C, YB, Br64f, LUNG | B70f, BI57d, BI57D\2, YB, C, LUNG | B, B31f, STANDARD, B_SHARP_C | B70f, I70f\1, FC56, LUNG | B_SHARP_C, SB, I30f\3, Br40, LUNG |

**Table S2 Baseline characteristic of patients with NSE**

| Characteristic | Training Set  (n=161) | | P value | External Test Set  (n=93) | | P value |
| --- | --- | --- | --- | --- | --- | --- |
|  | LNEN  (n=75) | LADC  (n=86) |  | LNEN  (n=27) | LADC  (n=66) |  |
| Age (y) ^✝^ | 65 (60.0, 69.0) | 65 (59.0, 69.3) | .536 | 60 (55.0, 66.0) | 58 (53.8, 65.0) | .406 |
| Sex (male) | 63 (84.0) | 69 (80.2) | .535 | 19 (70.3) | 38 (57.6） | .250 |
| NSE (≥16.3ng/ml） | 15 (20.0) | 6 (7.0) | .014^*^ | 11 (40.7) | 19 (28.8) | .263 |

Note: Unless otherwise indicated, data are numbers of patients, and data in parentheses are percentages. LNEN = lung neuroendocrine neoplasms, LADC = lung adenocarcinoma, NSE = neuron-specific enolase.

^✝^ Data are medians, with interquartile ranges in parentheses.

^*^ P-values are statistically significant.

**Table S3 Comparison of baseline patient characteristics between the training set and external test set**

| Characteristic | Training Set  (n=316) | External Test Set  (n=129) | P value |  |
| --- | --- | --- | --- | --- |
| Age (y) ^✝^ | 65.0 (60.0, 69.0) | 60.0 (54.0, 67.0) | <.001^*^ |  |
| Sex (male） | 262 (82.9) | 83 (64.3) | <.001^*^ |  |
| Outer 1/3 lung zone (present) | 181 (57.2) | 63 (48.8) | .104 |  |
| RU (present) | 96 (30.4) | 40 (31.0) | .896 |  |
| Lobulation (present) | 296 (93.7) | 123 (95.3) | .494 |  |
| Spiculation (present) | 111 (35.1) | 49 (38.0) | .569 |  |
| Pleural indentation (present) | 97 (30.7) | 44 (34.1) | .483 |  |
| Air bronchogram (present) | 22 (7.0) | 12 (9.3) | .399 |  |
| Vascular convergence sign (present) | 115 (36.4) | 42 (32.6) | .442 |  |

Note: Unless otherwise indicated, data are numbers of patients, and data in parentheses are percentages. RU = upper lobe of right lung.

^✝^ Data are medians, with interquartile ranges in parentheses.

^*^ P-values are statistically significant.
